# Interindividual Variation in the Proteome of Human Peripheral Blood Mononuclear Cells

**DOI:** 10.1371/journal.pone.0061933

**Published:** 2013-04-11

**Authors:** Evelyne Maes, Bart Landuyt, Inge Mertens, Liliane Schoofs

**Affiliations:** 1 Research Group of Functional Genomics and Proteomics, KU Leuven, Leuven, Belgium; 2 Flemish Institute for Technological Research (VITO), Mol, Belgium; 3 CFP/CeProMa, Antwerp, Belgium; University Medical Center Utrecht, The Netherlands

## Abstract

Peripheral blood mononuclear cells (PBMCs) are main actors in inflammatory processes and linked to many diseases, including rheumatoid arthritis, atherosclerosis, asthma, HIV and cancer. Moreover, they seem an interesting ‘surrogate tissue’ that can be used in biomarker discovery. In order to get a good experimental design for quantitative expression studies, the knowledge of the interindividual variation is an essential part. Therefore, PBMCs were isolated from 24 healthy volunteers (15 males, 9 females, ages 63–86) with no clinical signs of inflammation. The extracted proteins were separated using the two dimensional difference in gel electrophoresis technology (2D-DIGE), and the gel images were processed with the DeCyder 2D software. Protein spots present in at least 22 out of 24 healthy volunteers were selected for further statistical analysis. Determination of the coefficient of variation (CV) of the normalized spot volume values of these proteins, reveals that the total variation of the PBMC proteome varies between 12,99% to 148,45%, with a mean value of 28%. A supplemental look at the causes of technical variation showed that the isolation of PBMCs from whole blood is the factor which influences the experimental variance the most. This isolation should be handled with extra care and an additional washing step would be beneficial. Knowing the extent of variation, we show that at least 10 independent samples per group are needed to obtain statistical powerful data. This study demonstrates the importance of considering variance of a human population for a good experimental design for future protein profiling or biomarker studies.

## Introduction

Proteins are important effector molecules and changes between different biological states (e.g. healthy and sick) can be characterized by alterations in protein abundances. For this reason, the analysis of the proteome is one of the major paths to discover biomarkers which enable early diagnosis and improve treatment of several diseases. Although major efforts have been accomplished the last fifteen years and a large number of biomarker candidates have been listed, only a few biomarkers have been approved by the FDA [1]. One of the reasons why proteomics cannot yet fulfill the promise of rapid progress in biomarker discovery, is the ignorance of the variance among different samples and the thereby used proper experimental design [2].

A good experimental design means that reliable statistical results are obtained, which is reflected by the power of the statistical test. This power is the ability to detect an effect, if the effect exists, and depends on following parameters: significance level, effect size ( = fold change), sample size and variance [3]. For differential proteomics experiments, it is thus necessary to know and quantify the sources of variation and to limit them as much as possible [4]. This interindividual variation can be the result of both biological factors like gender, age, health status and environment as well as of methodological issues such as sample preparation or the analytical identification process. Next, also sample size is an important issue to be considered [3]. Suboptimal amounts of samples will not give significant valuable results, but on the other hand, oversampling is a waste of materials, money and time. The amount of variation can have its implications in sample size number required for a study, as samples with high variance values require more biological replicates than samples with a low variation to generate meaningful statistical results [5].

While plasma or serum samples are the most explored tissues in biomarker discovery, peripheral blood mononuclear cells (PBMCs) have potential as non-invasive matrix as well. This heterogeneous cell population consists out of monocytes and lymphocytes, and is the main actor in inflammatory processes. Understanding the changeability of the genome and proteome of these blood cells, can provide new insights in the function of PBMC in many pathologies. For this reason, PBMC cell fractions were already used for several genomics and proteomics studies, which are described elsewhere [6]–[13]. All these studies indicate that PBMCs are an interesting ‘surrogate tissue’ to search for biomarkers.

In this study, we will determine the extent of interindividual variation in the PBMC proteome of healthy volunteers using a gel-based proteomic approach. Although the analytical variance of the PBMC fraction using 2D gel electrophoresis within and between laboratories is already described [14], we use the 2D-DIGE approach, which is more common nowadays for differential proteomic analysis. Moreover, we focus in this study on the interindividual variance as the proteome of 24 elderly healthy volunteers is compared. We will also establish the percentage of high variable proteins and look into the factors that contribute to the technical variation in our experiments. Next, the sample size for experiments using a 2D-DIGE approach with PBMC fractions is determined. This way, an appropriate setup can be proposed for future 2D-DIGE discovery experiments using the PBMCs.

## Materials and Methods

### Ethics statement

The blood samples were taken with the approval of the local ethical committee (Clinical Trial Center, UZ Leuven, Campus Gasthuisberg, ML6505) and a signed informed consent from every volunteer is available.

### PBMC sampling

Blood from 24 healthy volunteers (15 males, 9 females, ages 65–86, with no clinical signs of inflammation)(Table 1) was collected in 4×1.8 ml 0,109 M buffered sodium citrate vacutainers (Venosafe, VWR, Leuven, Belgium) and were processed within 4 hours after blood withdrawal. For the isolation of the PBMC cells, leucosep tubes (Greiner Bio-One, Wemmel, Belgium) were used. Blood was diluted 1∶1 with Dulbecco's Phosphate Buffered Saline (PBS)(Sigma, St Louis, Missouri) prior to transferring it into the leucosep tube. After centrifugation (10 min, 1000 g and ambient temperature), the PBMC cell layer of two leucosep tubes were pooled and transferred into a 15 ml falcon. To wash the PBMCs, the sample was diluted with 10 ml PBS and centrifuged again for 10 min at 250 g and ambient temperature. The obtained cell pellet was resuspended in 10 ml PBS, to wash the cells a second time. After centrifugation (10 min, 250 g, ambient temperature), the cell pellet was stored at −80°C until further analysis.

**Table 1:** Characteristics of healthy volunteers.

Nr	Gender	Age	Lifestyle	CRP*	BMI	Sedimentation^§^	Leukocytes^#^	Lymfocytes (%)	Monocytes (%)	
1	M	69	non-smoker	0	27,68	15	6600	27,8		11,9
2	M	73	non-smoker	0	26,12	17	7240	38,4		7,9
3	F	67	non-smoker	0	27,55	39	8890	38,1		7,5
4	M	73	ex-smoker	0	24,91	8	3890	18,8		10,8
5	F	69	ex-smoker	0	27,55	30	7030	38		7,4
6	F	76	non-smoker	0	22,5	25	6130	18,8		7,7
7	M	71	ex-smoker	0	31,02	21	8230	32,4		7
8	M	67	non-smoker	0	22,86	3	4870	28,1		4,7
9	M	77	ex-smoker	0	29,38	4	6750	21,9		8,6
10	F	74	non-smoker	0	29,38	23	5310	35,8		9,8
11	M	74	ex-smoker	0,1	25,71	12	11600	12,2		6,6
12	F	71	smoker	0	32,88	7	8880	28,4		6,9
13	F	67	ex-smoker	0,3	31,25	49	9330	36,3		10,1
14	M	81	non-smoker	0,1	31,89	4	4040	32,7		5
15	M	73	ex-smoker	0	23,78	27	6240	29,6		8,5
16	M	73	ex-smoker	0,1	24,69	30	6950	28,9		10,8
17	M	76	ex-smoker	<0,5	24,5	5	4550	14,3		6,2
18	F	75	non-smoker	<0,5	22,31	2	4030	31		8,7
19	M	67	ex-smoker	<0,5	26,42	5	5850	20,5		7,7
20	F	63	non-smoker	<0,5	30,12	/	4220	37		17,3
21	M	68	ex-smoker	<0,5	30,93	14	7430	41,2		9
22	F	67	non-smoker	<0,5	29,41	11	4710	22,7		8,9
23	M	86	ex-smoker	<0,5	22,49	19	5520	22,3		10,7
24	M	79	ex-smoker	<0,5	24,28	10	5290	30,1		11,3

* CRP  =  C-reactive protein (mg/dl), ^§^Sedimentation after 1 hour (mm),^#^Number of leukocytes/µl.

### Sample preparation

Cell pellets were resuspended in 500 µl lysis buffer (7 M urea, 2 M thiourea, 4% chaps, 40 mM tris-base, 1% dithiothreitol (DTT)) with 1% protease inhibitor. After sonication of the samples, they were concentrated using Amicon Ultra 4 Centrifugation filters (10 kDa) (Millipore, Brussels, Belgium). The resulting sample (ca. 150 µl) was desalted via dialysis for 2 hours on 4°C (1 kDa cut-off, GE Healthcare, Freiberg Germany). Next, the concentration of the proteins was determined using the Bradford method and afterwards, the pH of each sample was measured.

### 2D-DIGE

For each sample, 50 µg of proteins was labeled with 400 pmol of either Cy3 or Cy5, using minimal labeling (GE Healthcare). An internal standard of all samples was prepared by pooling 25 µg of each sample and after aliquoting this pool in 12 samples, they were labeled with 400 pmol of the Cy2 fluorophore. The labeling was performed in the dark and on ice during 30 minutes. The reaction was stopped by adding 10 mM lysine and the samples were stored on ice for 15 minutes. After pooling the Cy2, Cy3 and Cy5 sample for each gel, the first dimension was initiated.

The labeled proteins were separated in a first dimension using Immobilized pH gradient (IPG) strips (NL, pH 3–10, 24 cm) (GE Healthcare), which were rehydrated the day before with Destreak reagent containing IPG buffer. Iso-electric focusing was performed on an IPGphor II (GE Healthcare) until 50,000 VHrs were reached. The settings of the first dimension were as follows: 3 h at 150 V in step-n-hold, 3 h at 300 V in step-n-hold, 6 h at 1000 V in gradient, 3 h at 8000 V in gradient, 3 h at 8000 V in step-n-hold. Before starting the second dimension, the strips were first equilibrated by soaking them in sodium dodecyl sulphate (SDS) equilibration buffer (50 mM tris Cl pH 8.8, 6 M urea, 30% glycerol, 2% SDS) with 1% DTT. After 15 min, the strips were placed in SDS equilibration buffer with 4% iodoacetamide and bromophenolblue, which was added as tracking dye.

Spotpickgels were created by using Bind-Saline working solution (12 ml ethanol, 300 µl acetic acid, 15 µl bind-silane, 2.7 ml MilliQ) to stick the gel to the plate.

The strips were then added on the SDS-PAGE gels (12%), and covered with agarose. The second dimension was carried out with an Ettan DIGE Twelve electrophoresis system (GE Healthcare) with following parameters: 600 V, 8 mA/gel and 10 ω. After one hour, the settings were changed to 12 mA/gel. The second dimension was stopped after 20 h and the gels were scanned using the Ettan DIGE imager. The resolution was set at 100 µm, and the scanning exposure time was optimized for every gel, to prevent saturation of interesting protein spots. After scanning the gel, total protein content was visualized by Deep purple staining. In short, gels were fixated overnight in 10% methanol, 7.5% acetic acid. The next day, the gels were washed and then stained with deep purple dye for 1 h. After 2 washing steps with 7.5% acetic acid, the gels were scanned at 590 nm.

### Image analysis

The gel images were loaded into the Decyder 2D 7.0 software (GE Healthcare). In the Differential In Gel Analysis module, settings for optimal intra-gel spot detection were determined. The estimated number of spots was set on 10,000, but the protein spots were filtered according to their volume (greater than 40,000 pixels) to prevent dust particles to be seen as spots. Next, the Cy2, Cy3 and Cy5 gel images were merged and normalized spot volumes were calculated. The processed gels were then loaded into the biological variation analysis tool, a master gel was chosen and all 36 gel images were matched. According to the manufacturer's protocol, manual detection of the spotmatching was done using landmarking and re-matching. The coordinates of the spots of interest were loaded into a picklist for the Ettan Spotpicker and spots were automatically excised.

### Protein digestion and mass spectrometry

The excised spots were washed twice with 50 µl MilliQ, followed by 3×50 µl acetonitrile. After three cycles of hydration with acetonitrile and rehydration with 100 mM ammonium bicarbonate, the gel pieces were vacuum dried in a vacuum concentrator. To start the enzymatic digestion, 25 µl of a solution containing 5 ng/µl trypsin (Promega, Fitchburg, WI), 50 mM ammonium bicarbonate and 5 mM calciumchloride was added to each gel piece and placed on 37°C overnight. The next day, the tryptic peptides were extracted using 50 mM ammonium bicarbonate followed by an extraction with 50% acetonitrile and 5% formic acid. This step was repeated twice. Afterwards, the pooled extracts were vacuum dried and the peptides were stored at −20°C. Prior to mass spectrometric analysis, the samples were desalted and concentrated using C18 ZipTips (Millipore) according to the manufacturer's instructions. One µl of every desalted sample was spotted on a stainless steel target plate, and every sample was covered with 1 µl of saturated alfa-cyano-hydroxy-cinnacid acid dissolved in 50% acetonitrile and 0.1% formic acid. Spots were analyzed using an Ultraflex II Matrix Assisted Laser Desorption/ionization Time-of-flight (MALDI-TOF) (Bruker Daltonics, Bremen, Germany). The spectra were measured using a positive ion reflectron mode. The peptide calibration standard (Brucker Daltonics) contained nine standard peptides, including bradykinin (757.3992 Da), Angiotensin II (1046.5418 Da), angiotensin I (1296.6848 Da), Substance P (1347.7354 Da), Bombesin (1619.8223 Da), Renin substrate (1758.9326 Da), ACTH clip 1–17 (2093.0862 Da), ACTH clip 18–39 (2465.1983 Da), and somatostatin 28 (3147.4710 Da).

For every spot, a total of 3600 shots were summed. Spectra were analyzed using Biotools MS software to perform peptide mass fingerprinting using the in-house MASCOT server (Matrix science, London, U.K.). For the identification of the proteins, the SwissProt database was used, specified for *homo sapiens* taxonomy and with a carbamidomethyl on cystein set as fixed modification and a methionine oxidation as variable modification. We specified the use of trypsin as digestion enzyme. Peptide tolerance was set at 0.2 Da and the decoy function was used. Protein identifications with a Mowse score greater than 56 were considered as significant (p<0.05).

### Statistical analysis

In the BVA module of the Decyder 2D 7.0 software, the standard abundance (SA) for each spot was reported as the ratio of the spotvolume of Cy3 (or Cy5) to the volume of the Cy2 standard. Standardized log abundance (SLA) values were used to quantify the differential expression. Only protein spots appearing in at least 11 out of 12 gels were used for statistical analysis. After exporting the raw data of the proteins of interest, further statistical processing of the spot characteristics was performed in Excel and R. Spotwise standard deviations (SD), arithmetic mean (µ) and coefficient of variation (CV) values of the SA values were calculated for each spot. For the determination of sample size and statistical power, PASS 11 software (NNCS, Utah, USA) was used. Results were obtained by inequality tests for two means using ratios (two sample t-test), as is also used by the DeCyder software.

## Results

We analyzed the interindividual variation in the PBMC proteome in a heterogeneous elderly healthy control population. This variation is the result of both biological and technical factors. To evaluate the total variation, we applied a difference in gel electrophoresis experiment of PBMC fractions of 24 healthy volunteers, aged over 60 years, with no clinical signs of inflammation (Table 1). In order to detect the maximum number of proteins, non linear IPG strips with a pH range from 3–10 and 12% PAGE gels were used to visualize proteins between 20 kDa and 200 kDa. To mimic the setup used in differential experiments, two healthy volunteer samples were matched according to gender and age, and labeled with either Cy3 or Cy5. Also, an internal standard, labeled with Cy2, was used for normalization. The experimental setup can be found in Table 2. After electrophoresis and scanning of the gels, the gel images were loaded in DeCyder 2D 7.0 software and an extensive matching, re-matching and landmarking was conducted. In total, up to 2513 spots were detected on the gels.

**Table 2:** Experimental setup of total variation experiment: The samples on one gel are matched according to age and gender.

	Cy3	Cy5	Cy2		Cy3	Cy5	Cy2
Gel 1	HV 1	HV 21	pool	Gel 7	HV 22	HV 20	pool
Gel 2	HV 2	HV 15	pool	Gel 8	HV 10	HV 18	pool
Gel 3	HV 3	HV 13	pool	Gel 9	HV 6	HV 24	pool
Gel 4	HV 12	HV 5	pool	Gel 10	HV 14	HV 23	pool
Gel 5	HV 7	HV 11	pool	Gel 11	HV 19	HV 8	pool
Gel 6	HV 16	HV 4	pool	Gel 12	HV 9	HV 17	pool

HV = healthy volunteer.

**Figure 1:**
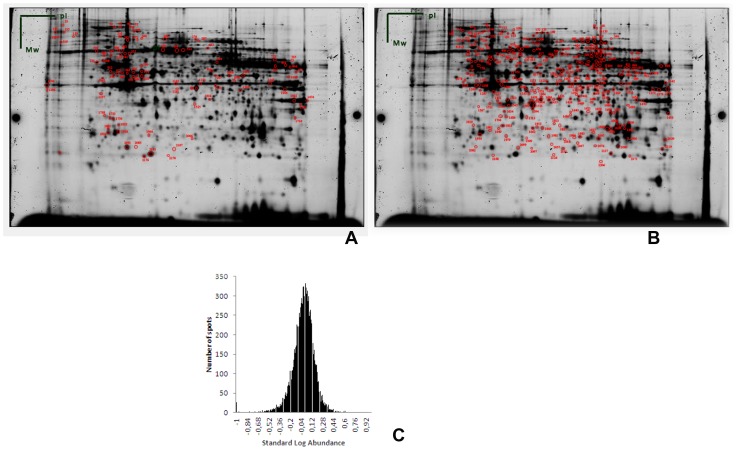
Overview of high quality protein spots used for statistical analysis. Deep purple stained gels with overview of high quality spots present in all gels (A) and 11 out of 12 gels (B). Panel C shows the standard Log abundance values of all high quality protein spots in at least 11 out of 12 gels (C). The Gaussian distribution of the SLA values confirms regular data.

Although all protein spots from a 2D-DIGE experiment can be of interest, we chose to work with spots present in at least 11 out of 12 gels, as these spots are able to give a better estimation (more samples, more volume ratios, better statistical relevance) of the variance in this experiment. Furthermore, we assume that the biological and technical concepts discussed, can be extended to all spots on the gel. The highly reproducible protein spots used for the estimation of total variation are shown in Figure 1.

After extraction of the raw data, we calculated the CV of 382 spots using the Vnorm_g_ values. These normalized values represent the standard log abundance (SLA) values, which gives the ratio of Cy3/Cy2 or Cy5/Cy2. As shown in Figure 1C, the Gaussian distribution of the SLA values confirms regular data. After making a pair wise comparison of the spots in the DeCyder software using t-test statistics combined with FDR correction, none of the spots turned out to be a false positive differential protein.

**Figure 2:**
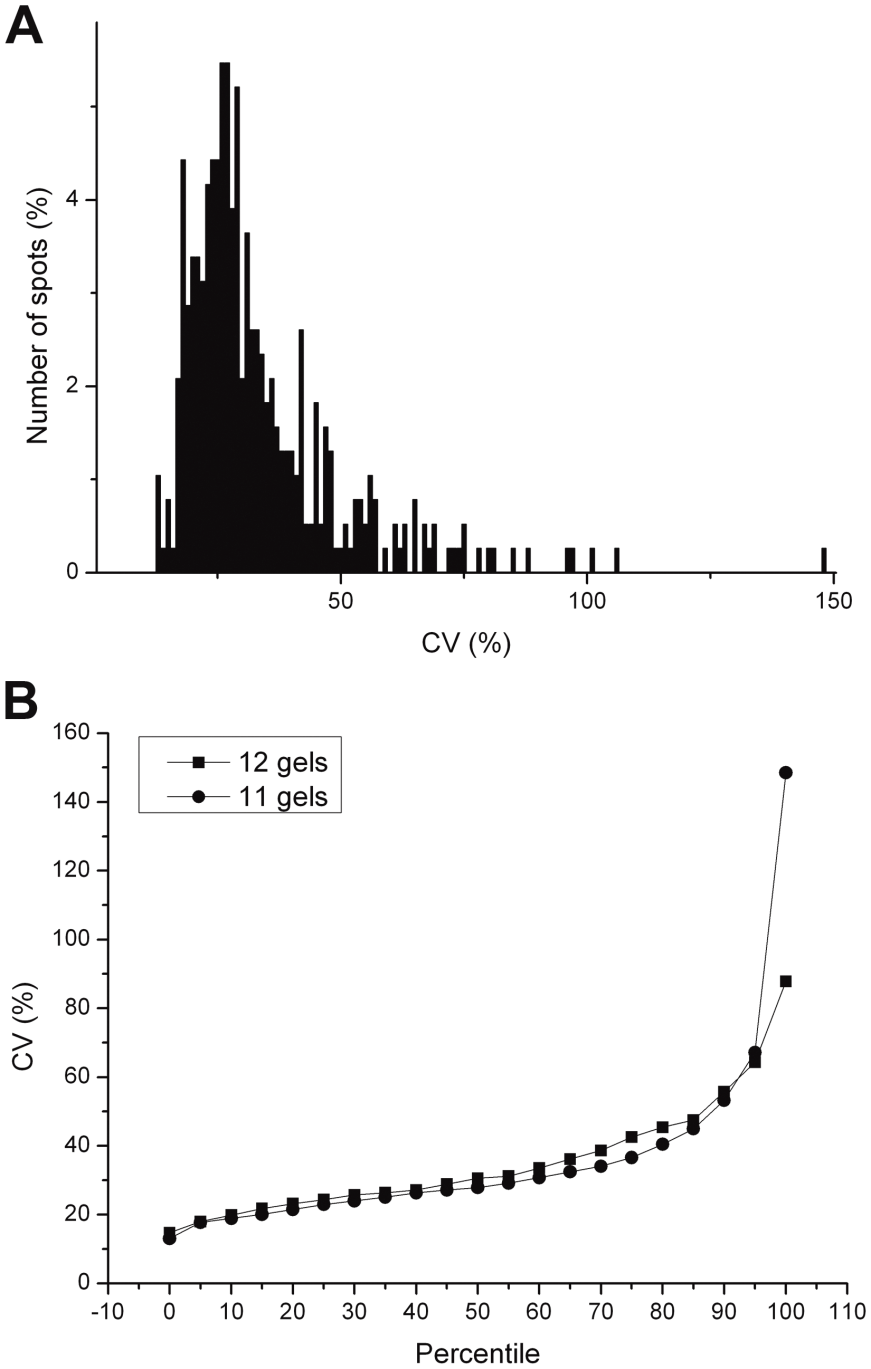
Distribution of total variation data. Distribution of CV values of high quality spots in at least 11 out of 12 gels (A). The CV values of 2 different subsets of high quality spots (B). The curves represents the spots matched in all 12 gels (111 spots) and 11 out of 12 gels (273 spots).

To have an idea about the spotwise variation of the selected proteins in this cell fraction, the coefficient of variation for every spot was calculated, using the standard abundance values. The CV of these spots ranged from 12,99% to 148,45%, with a mean value of 28%, as can be seen in Figure 2. Consequently, the interindividual variation in these mononuclear blood cells varies about 28%. Up to 75% of the spots do not exceed the CV value of 40%, which shows that most of the protein abundances are quite stable in 24 healthy individuals. Proteins exceeding the threshold of CV = 50%, are highly variable proteins, and cannot be used in differential biomarker discovery procedures, because their interindividual variation limits the detection of true biologically significant differences. Only 13% of all the protein spots used, turned out to be highly variable proteins. These proteins were selected for identification by MALDI-TOF mass spectrometry (Table 3).

**Table 3:** List of high variable proteins identified via MALDI-TOF Mass spectrometry.

Nr	Name	ID	Score*	Coverage^§^	peptides^¥^	pI^#^	Mw^§§^	CV^##^
302	Ezrin	EZRI_HUMAN	81	27	19	5,94	69486	0,69
306	Zyxin	ZYX_HUMAN	62	23	8	6,22	62456	0,75
307	Ankyrin repeat domain-containing protein 60	ANR60_HUMAN	58	35	10	9,2	38070	0,85
317	Aldehyde dehydrogenase family 16 member A1	A16A1_HUMAN	63	20	13	6,35	86118	0,81
459	Serum albumin	ALBU_HUMAN	95	23	18	5,92	71352	0,63
491	Serum albumin	ALBU_HUMAN	121	33	24	5,92	71352	0,69
513	Serum albumin	ALBU_HUMAN	123	34	27	5,92	71352	0,65
541	Plastin-2	PLSL_HUMAN	217	58	32	5,29	70824	0,57
545	Plastin-2	PLSL_HUMAN	214	50	32	5,29	70824	0,97
550	Plastin-2	PLSL_HUMAN	228	44	31	5,29	70824	0,54
726	Protein disulfide-isomerase	PDIA1_HUMAN	296	63	36	4,76	57487	0,8
742	Protein disulfide-isomerase A3	PDIA3_HUMAN	224	51	31	5,98	57153	0,51
846	ATP synthase subunit alpha, mitochondrial	ATPA_HUMAN	73	28	14	9,16	59830	0,68
918	Fibrinogen beta chain	FIBB_HUMAN	79	29	13	8,54	56588	0,54
971	Protein disulfide-isomerase A6	PDIA6_HUMAN	133	40	19	4,95	48497	0,53
	Dynactin subunit 2	DCTN2_HUMAN	83	40	13	5,1	44320	0,53
	ATP synthase subunit beta, mitochondrial	ATPB_HUMAN	75	36	14	5,26	56525	0,53
1123	Actin, cytoplasmic 1	ACTB_HUMAN	130	52	18	5,29	42058	0,75
1708	Annexin A5	ANXA5_HUMAN	68	25	9	4,94	35972	0,56
2091	Apolipoprotein A-I	APOA1_HUMAN	82	32	9	5,56	30759	0,56
2095	Phosphatidylethanolamine-binding protein 1	PEBP1_HUMAN	78	48	10	7,01	21160	0,74
								

*: Mascot probability based Mowse score: Score = −10log p, where p is the absolute probability that the given hit is a random event. Significance (p<0.05) is reached at scores >55. §: Sequence coverage in % of amino acid sequence ¥: Number of peptides matched to whole protein §§: Theoretical value of isoelectric point #: Theoretical value of molecular weight ##: coefficient of variation

To get a better idea about the distribution of the CVs, the data were divided in two subsets. One set comprises the spots matched in all the gels, a second subset includes all the spots matched in 11 out of 12 gels. As seen in Figure 2B, the subsets both show the same pattern of CVs but diverge at the 95^th^ percentile. The CV values of 12 gels between the 95^th^ and 100^th^ percentile are ranging between 64,24% and 87,78%. On the other hand, the CVs of proteins out of 11 gels have values between 67,12% en 148,45%, which indicates the presence of some outliers.

Because outliers were seen and in order to try to control this variation, an extra experiment was performed to determine which factors of the technical variation could be responsible. As this technical variation can be split up in the variation due to the electrophoresis and CyDye labeling and the variance induced by sample preparation, two separate tests were performed. The setup of this experiment is shown in Table 4.

**Table 4:** Experimental setup for technical variation experiment.

Electrophoresis + Labeling	Cy3	Cy5	Cy2
Gel 1	Sample 1	Sample 1	Sample 1
Gel 2	Sample 2	Sample 2	Sample 2
Gel 3	Sample 3	Sample 3	Sample 3
**Sample preparation**	**Cy3**	**Cy5**	**Cy2**
Gel 4	Sample 4	Sample 5	Pool
Gel 5	Sample 6	Sample 7	Pool
Gel 6	Sample 8	Sample 9	Pool

**Figure 3:**
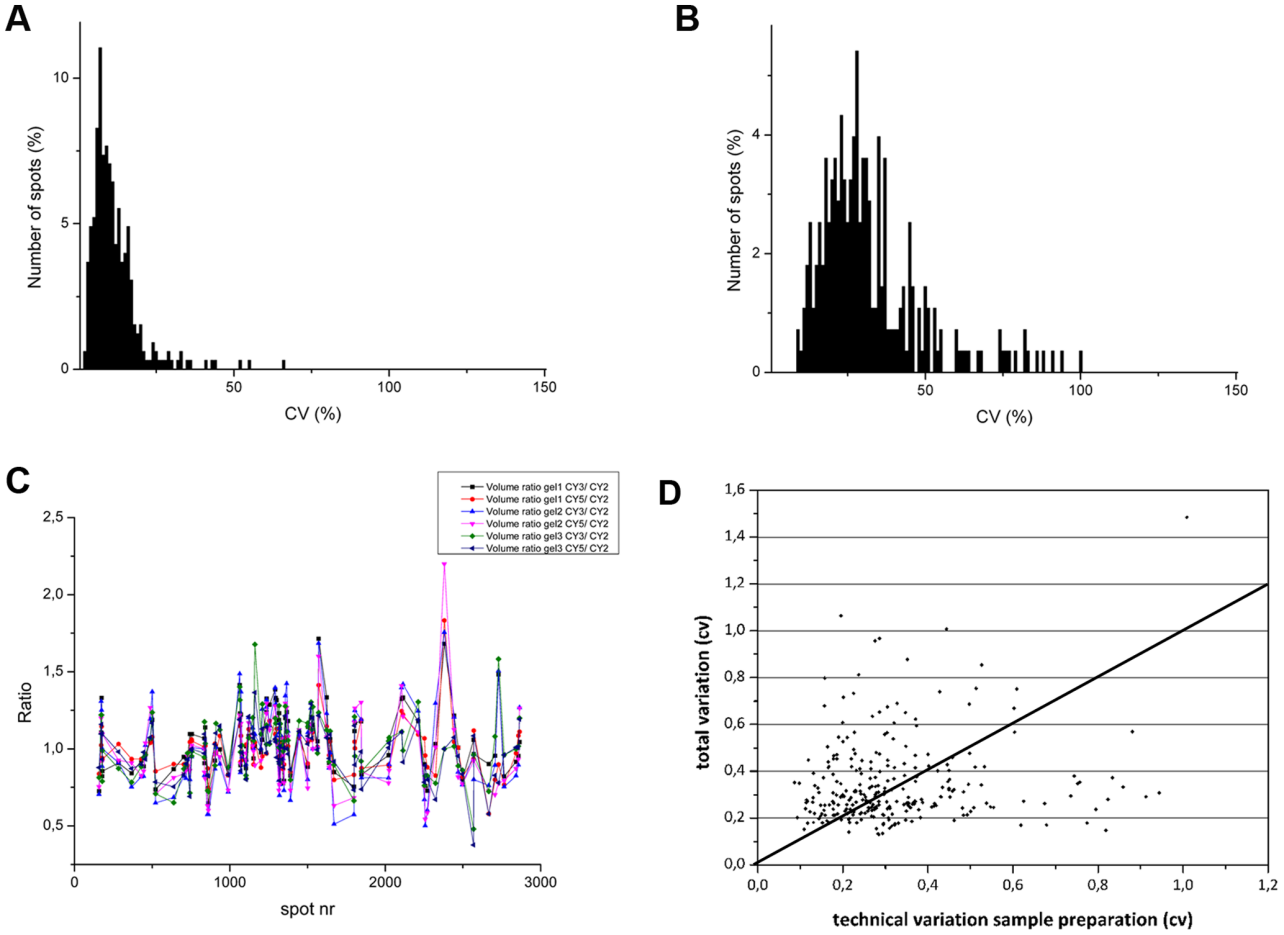
Overview of technical variation. Panel A shows the technical variation due to Cydye labeling and the electrophoresis process. Panel B shows the technical variance due to sample preparation. Panel C shows the deviation of the Cydye ratio. Ideally, the ratio of Cy3 or Cy5 versus Cy2 should be equal to 1. Any deviation can be directed to technical variation (A). Panel D illustrates the contribution of technical issues of sample preparation to the total variation. The scatter plot indicates that this technical variation has a major role in the total variance.

In the first test, blood from a single withdrawal was processed and subsequently divided in three samples. Afterwards, these samples were labeled with Cy2, Cy3 or Cy5, mixed together and run on one gel. This experiment was performed in triplicate. Theoretically, the Cy3/Cy2 or Cy5/Cy2 standard abundance ratios should be 1, and any deviation can be directed to variation in this process. For all the proteins of interest, these deviations can be seen in Figure 3C. To further quantify this variation, the CV of the SA values of the six ratios was calculated. The average CV value of the electrophoresis and labeling is 11.75%, ranging from 2% until 65%. Most of the proteins (92%) demonstrate a CV below 20% (Figure 3A).

In the second test, the variance with regard to sample preparation was determined. Consequently, six distinct PBMC isolation procedures from a single blood withdrawal were obtained. Three samples were labeled with the Cy3 fluorophore, three with Cy5 and a pool of all samples was created and labeled with the Cy2 dye. Next, 3 gels were run and the CV values were determined. The average CV from the sample preparation was 32.05% with outliers until CV = 100% (Figure 3B). To evaluate the proportion of this technical variation due to sample preparation to the total variation, both CV values were plotted against each other (Figure 3D).Several spots do show that the technical variation is the major contribution to the overall variation of the protein.

**Figure 4:**
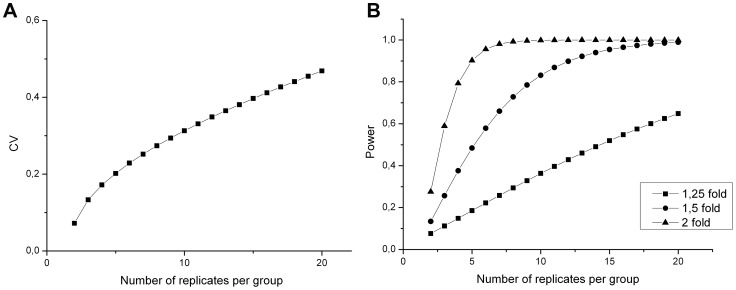
Influence of coefficient of variation. Panel A shows the influence of coefficient of variation on sample size, assuming a power of 0,8 and a fold change of 1,5. The higher the variation in a setup, the more replicates are needed to obtain the same power. Panel B illustrates power versus number of replicates when detecting various fold changes with following parameters: CV = 30% and a significance level of 0,05. The more subtile changes one wants to observe, the more replicates are required (B).

Finally, we calculated the sample size which is needed to achieve statistically reliable results in biomarker discovery using PASS software (Figure 4). In order to detect true differences with a fold change of 1,5, following settings were used: statistical power of 0.8 and significance level of 0,05 (Figure 4B).

## Discussion

Understanding the degree of variation among different samples is important for proteomics studies designed to detect true differences. Furthermore, it is also necessary to quantify the sources of variation linked to the proteomics method used, and to limit them to the best possible extent. In this study, we evaluated interindividual variations in peripheral blood mononuclear cells by analyzing 24 PBMC fractions from elderly healthy volunteers, using 2D-DIGE. A typical three dye setup was applied to reflect the system used in quantitative proteome analysis. We did chose to study elderly healthy volunteers, as incidences in several diseases linked to inflammation, including cancer, increase exponentially with advancing age. Moreover, it is known that the immune system of elderly persons differs from that of younger people in several aspects including the function, number and development of macrophages and lymphocytes [15], [16]. The proteome of this heterogenous population will more likely match better with the proteome of the population of cancer patients that is used for biomarker discovery. We know however, that using this heterogeneous population will increase our total variation, as both gender and age are important factors contributing to the biological variance.

Our results reveal that the variation in the PBMC proteome of an elderly control population ranged from 12,99% to 148,45%, with an average value of 28%. A comparison with other human variation studies, showed that our data were consistent with other CV values. A proteomic analysis of individual variation in normal human livers using difference gel electrophoresis revealed that the CV of spots detected in all 10 individuals ranged from 6,4% to 108,5% and the median CV was 19% [17]. Yamakawa and co-workers showed that the variation in the seminal plasma proteome of healthy fertile individuals ranged from 24,5% to 129,9%, with a median value of 63,1% [18]. The variation of the platelet proteome of 20 healthy volunteers, determined by 2D DIGE, varies about 18% [19]. Corzett and colleagues focused their research on the statistical analysis of the variation in the proteome of human plasma. For their study, samples were taken from 11 individuals at 3 time points. A median interindividual CV of 23% was found and the range of this spotwise variation was from 10% to 93% [20], [21]. A comparative analysis of the inter- and intraindividual variation in human cerebrospinal fluid, demonstrated also that the fluctuations in protein abundance within an individual are smaller than interindividual variation [22]. Stoop and colleagues examined the proteomic variation in this cerebrospinal fluid and found a total variance ranging between 18% and 148% [23].

In this study, 13% of the spots have a interindividual variation higher than 50%. Identification of these high variable proteins (Table 3) showed us that the identified proteins cannot be linked to one functional category, but comprises several classes like metabolic enzymes and cytoskeletal remodeling proteins. A comparison with the high variable proteins identified in monocytes purified from PBMCs [24], confirmed that some proteins like plastin are highly variable in a general control population. However, some identified proteins, like albumin, fibrinogen, apolipoprotein A–I and annexin 5, are known to be abundant plasma proteins and are probably artifacts from the PBMC isolation procedure. These proteins might stick to the membranes of the isolated PBMC cells and may not have been washed away sufficiently enough. In this way, their high difference in abundance between the different samples can be linked to the sample preparation procedure. For that reason, it would also be interesting to know if proteins are suffering more from the sample preparation procedure, and are thus showing a high technical variation.

In order to determine the technical variation, we performed two tests. As we were only interested in the variation values of the subset of protein spots used in the previous variation experiment, just these raw data were extracted. In the first test, the variance during labeling and electrophoresis was established. Herefore, a PBMC fraction from one healthy volunteer was subdivided in three samples, and each of them were labeled with Cy2, Cy3 or Cy5 respectively. After analysis of CV values of the spots, it seemed that the electrophoresis procedure and labeling is quite consistent during the whole experiment, as 92% of the proteins did have CV values below 20%.

In the past, de Roos *et al.* established the within and between laboratory variation in PBMCs using classical 2D gel electrophoresis. They found a technical variation (within laboratory) that ranged from 18% to 68% [14]. Through our results, we can confirm that with the use of an internal standard in the DIGE procedure leads to less technical variation than compared to classical 2D PAGE and that the data across the gels are more comparable [25]. Furthermore, by using automated spotpicking systems, the excision of protein spots of interest is more precise than the manual cutting. The variation due to electrophoresis and labeling of samples is thus only a very small fraction of the total variation and has reached the optimal conditions.

In a second part, the technical variance linked to the isolation of the blood cells and sample preparation was established. Herefore, six distinct PBMC isolation procedures from a single blood withdrawal were performed. Three samples were labeled with Cy3, three others with Cy5, and a pool of all 6 samples was used as internal standard and labeled with Cy2. Again, the same spots as in the previous procedure were used to calculate the CV values. After analysis, it seemed that some proteins showed a high CV (up to 99%), and thus, have a high technical variance. This indicates that sample preparation is the most crucial step in the whole procedure and has to be optimized as much as possible.

When analyzing the contribution of the technical issues due to sample preparation to the total variation (figure 3D), the majority of the spots with high total variance values, also showed a substantial contribution of technical CV. For example, the outlier with an overall variation of 148%, has a technical variation (sample preparation) of 100%. The majority of the protein spots are situated above the 45° line and thus have higher total variation values than technical variation issues. Some other protein spots which are positioned close to the 45° line, do show the same variance levels when analyzing different individuals (total CV) or the same individual (technical CV).

For that reason, the isolation of PBMCs should be handled with extra care. Sufficient wash steps are required to remove the plasma proteins that stick to the leukocyte cell membrane, in order to decrease the overall variance. Furthermore it is known that several sample preparation techniques, like protein depletion methods, create variation in different samples. Also enrichment methods, like ultracentrifugation, used in this study, probably create technical variation among samples and should be avoided, if possible, when doing quantitative proteomic analyses.

Whenever the overall variance is too high due to for example several enrichment methods, a pooled experimental design can be an option. However, it is important to notice that, whenever biological samples are pooled, no information is gathered for each individual apart, but only group characteristics can be found [26].

Finally, an estimation is made according to the minimum sample size which is needed to have significant results in a quantitative experiment. As shown in this study (Figure 4), variation can have a major implication on the sample size. This graph shows that, the higher the overall variation in a certain setup, the more samples are needed to achieve the statistical reliable results.

To minimize the number of false positives in further quantitative proteomic analysis, an unbiased design and an adequately power is needed [27]. This statistical power is influenced by the fold change, variability, sample size and significance level [4]. In future biomarker discovery studies, following parameters must be achieved: a significance level of 5%, and a minimum power of 80%. Based on the results obtained in this study (variance situated around 30%), at least 10 samples per condition should be used to achieve a statistical power of 0,8 with a fold change of 1,5. Whenever one wants to detect even more subtle changes (fold change 1,25) within the proteome, the sample size should even be extended.

For future proteomic experiments with the DIGE setup, a minimum of 10 human PMBC samples per group are needed, in order to get reliable quantitative results. In this way, it is likely that a good experimental design and consistent sample and data collection protocol mean that many of the identified putative biomarkers are real disease-related signals.
